# The biological function of extracellular vesicles in prostate cancer and their clinical application as diagnostic and prognostic biomarkers

**DOI:** 10.1007/s10555-024-10210-w

**Published:** 2024-09-24

**Authors:** Patrizia Limonta, Sara Marchesi, Gaia Giannitti, Lavinia Casati, Fabrizio Fontana

**Affiliations:** 1 Department of Pharmacological and Biomolecular Sciences “Rodolfo Paoletti”, Università Degli Studi Di Milano, Milan, Italy; 2 Department of Health Sciences, Università Degli Studi Di Milano, Milan, Italy

**Keywords:** Prostate cancer, Extracellular vesicles, Tumor microenvironment, Drug resistance, Diagnosis, Prognosis

## Abstract

Prostate cancer (PCa) is one of the most commonly diagnosed malignancies and main causes of cancer-related deaths worldwide. It is characterized by high heterogeneity, ranging from slow-growing tumor to metastatic disease. Since both therapy selection and outcome strongly rely on appropriate patient stratification, it is crucial to differentiate benign from more aggressive conditions using new and improved diagnostic and prognostic biomarkers. Extracellular vesicles (EVs) are membrane-coated particles carrying a specific biological cargo composed of nucleic acids, proteins, and metabolites. Here, we provide an overview of the role of EVs in PCa, focusing on both their biological function and clinical value. Specifically, we summarize the oncogenic role of EVs in mediating the interactions with PCa microenvironment as well as the horizontal transfer of metastatic traits and drug resistance between PCa cells. Furthermore, we discuss the potential usage of EVs as innovative tools for PCa diagnosis and prognosis.

## Introduction

Prostate cancer (PCa) is the second most commonly diagnosed malignancy and the fifth leading cause of cancer-related deaths among men worldwide [1]. Although most PCa patients are diagnosed with inert or slow-growing disease, it has been estimated that approximately 20% of men have a high-risk and potentially lethal tumor. Furthermore, PCa is characterized by high heterogeneity and can be subdivided into various clinical states that are treated with different therapeutic strategies. Indeed, active surveillance is generally preferred for patients with indolent disease, radiotherapy and partial/radical surgery are adopted for individuals with localized tumor, while aggressive cancers are usually managed with a combination of hormonal therapy (GnRH agonists/antagonists, androgen receptor antagonists, and androgen synthesis inhibitors) and chemotherapy (taxanes) [2, 3]. However, patients frequently develop drug insensitivity, especially castration resistance, resulting in poor therapeutic outcome, disease relapse, and dismal prognosis [4, 5]. In this context, there is an urgent need to find novel clinically valuable prognostic and diagnostic biomarkers for PCa, in order to predict and prevent tumor recurrence and therapy evasion.

Extracellular vesicles (EVs) are a family of lipid bilayer-encapsulated particles enclosing a cargo of different bioactive molecules, including mRNAs, miRNAs, and other non-coding RNAs (ncRNAs), DNA, proteins, and lipids. EVs are shed by cells into the extracellular space and are deeply involved in the cell-to-cell communication occurring in different biological and pathological processes [6]. Based on their size, biogenesis, mechanism of release, and molecular cargo, they are classified in small and large vesicles (sEVs and lEVs, also known as exosomes and microvesicles, respectively). However, according to the recommendations of the International Society for Extracellular Vesicles (ISEV), the term “EVs” is now generally used to refer to both these vesicular families [7]. In the last years, accumulating evidence has pointed out the key role of EVs in the growth and aggressiveness of different tumors, including PCa. Specifically, these vesicles have been reported to be deeply involved in the vicious bidirectional communication between cancer cells and their bystander cells (fibroblasts, adipocytes, infiltrating immune cells, and endothelial cells) in the tumor microenvironment (TME) [8]. Moreover, they have been shown to be responsible for the horizontal transfer of metastatic traits and chemoresistance, also regulating cell phenotypic plasticity through the induction of stem-like state, epithelial-to-mesenchymal transition (EMT), and neuroendocrine differentiation (NED) [9, 10]. Owing to these characteristics, EVs have been widely investigated as potential diagnostic and prognostic biomarkers of tumor development, progression, and treatment response [11, 12]. However, despite the increasing importance of the extracellular vesicular compartment in PCa management, in-depth translational reviews about this topic are still scanty. In the present article, we aim to discuss the biological activities exerted by EVs during PCa evolution, specifically focusing on their involvement in the cell-to-cell communication in the TME and in the lateral transmission of malignant behavior. We also address their potential as novel diagnostic and prognostic biomarkers. Overall, we provide a bench-to-bedside overview of current literature about PCa vesicles.

## Biological roles of EVs in the cell-to-cell communication within PCa microenvironment

**Fig. 1:**
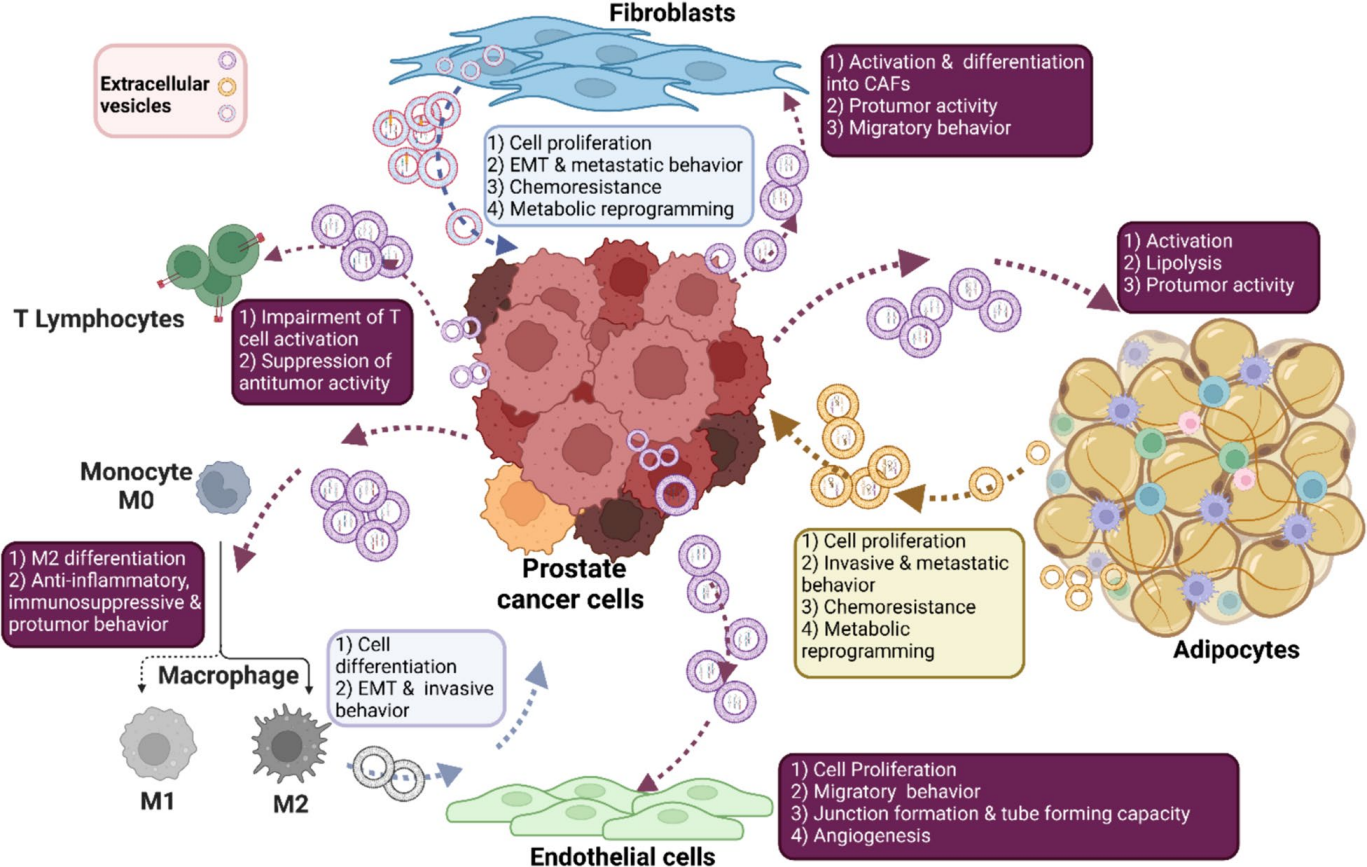
Schematic representation of the biological functions of EVs in the cell-to-cell bidirectional communication within the tumor microenvironment (TME) in prostate cancer (PCa). PCa cell-secreted EVs deliver their molecular cargo to neighboring cells (fibroblasts, adipocytes, immune cells, endothelial cells) to create a tumor-favoring microenvironment. Conversely, TME-derived EVs are taken up by PCa cells to foster their proliferation and invasion and to confer them resistance to conventional anticancer drugs

The key role of EVs in PCa progression and metastasis is now well-established. From the biological point of view, PCa EVs have been shown to deliver their molecular cargo to neighboring cells to create a tumor-sustaining microenvironment. On the other hand, TME-derived EVs are taken up by PCa cells to support their proliferative and invasive demands and to confer resistance to anticancer drugs (Fig. 1).

### PCa-fibroblast interplay

Cancer-associated fibroblasts (CAFs) are a crucial component of PCa stroma. They generally result from the recruitment and activation of different cell types, such as resident fibroblasts, vascular smooth muscle cells, and tumor-infiltrating mesenchymal stem cells, and are characterized by the expression of specific molecular markers, such as α-smooth muscle actin (α-SMA), fibroblast activating protein (FAP), and fibroblast-specific protein-1 (FSP-1); their induction is highly dependent on transforming growth factor-β (TGF-β) [13]. Interestingly, it is now well-known that PCa cells can corrupt stromal fibroblasts via EV production. Indeed, they have been found to release different vesicular subtypes enriched with TGF-β1 and 2, which trigger fibroblast protumoral transformation through the Smad2/3 and p38 signaling pathways [14, 15]; of note, this molecular cargo has been observed to be particularly abundant under hypoxic conditions [16, 17]. Similarly, high levels of hyaluronidase 1 (Hyal1), an enzyme involved in hyaluronic acid turnover and responsible for increased PCa cell motility, have been detected in tumor sEVs [18]; treatment of prostate fibroblasts with these particles does not affect their proliferation but significantly fosters their migratory behavior through the engagement of the focal adhesion kinase (FAK)-mediated integrin cascade [19]. Finally, it has been recently demonstrated that vesicles from PCa bulk and stem cells, a small cellular subpopulation characterized by self-renewal, differentiation potential, and cancer-initiating capacities [20], display a differential miRNA cargo that might cooperate in educating tumor stroma toward a cancer-favorable microenvironment; in particular, miR100-5p (overexpressed in all sEVs), miR21-5p (overexpressed in bulk cell sEVs), and miR139-5p (overexpressed in stem cell sEVs) are able to activate matrix metalloproteinases (MMPs) in normal fibroblasts, enhancing their invasive potential [21].

Given the bidirectional nature of the cell-to-cell communication occurring in the TME, studies have also been performed to investigate whether CAF-secreted EVs might affect PCa aggressiveness. For example, Dr. Zhao’s group has highlighted that sEVs shed by PCa patient-explanted CAFs trigger a metabolic reprogramming in tumor cells by inhibiting mitochondrial oxidative phosphorylation and increasing glycolysis; specifically, these vesicles are able to transfer their molecular cargo, composed of lipids, amino acids, and intermediates of the tricarboxylic acid cycle, to adjacent malignant cells, that in turn take advantage of these metabolites as substrates for energy and biomass generation under nutrient deficiency conditions [22]. Likewise, by means of *in vitro* and *in vivo* studies, Josson and coworkers have found that CAFs can release miR-409-enriched sEVs to promote PCa cell growth, EMT, and stemness [23]. In line with these data, sEV-packaged miR-1290 from transformed fibroblasts has been reported to stimulate castration-resistant PCa cell proliferation and migration through inhibition of the glycogen kinase synthase 3β (GSK3β)/β-catenin axis [24]. Notably, miR-27a and miR-423-5p shuttled by CAF-derived small vesicles have been shown to play a key role in the development of PCa resistance to standard chemotherapeutic drugs, through the modulation of the p53 and bone morphogenetic protein antagonist Gremlin 2 (GREM2)/TGF-β pathways [25, 26]. Conversely, Zhang and colleagues have observed that miR-146a-5p is downregulated in sEVs obtained from CAFs after hormonal therapy, contributing to the acquisition of a metastatic phenotype by recipient PCa cells through the activation of the epidermal growth factor receptor (EGFR)/extracellular signal regulator kinase (ERK) signaling [27]. More recently, small vesicles from normal human fibroblasts have been demonstrated to contain an elevated concentration of miR-3121-3p, which positively regulates the expression of the tumor suppressor NKX3-1 in androgen-sensitive PCa cells; this suggests that vesicular miR-3121-3p may play a role in preventing the conversion of hormone-dependent tumor cells toward a less differentiated state [28].

Taken together, the above results not only elucidate the molecular mechanisms underlying CAF formation but clearly evidence the wide range of effects that EVs exert in PCa-fibroblast communication.

### PCa-adipocyte interplay

Obesity has been shown to positively correlate with PCa aggressiveness, recurrence, and increased mortality [29]. In particular, adipocytes have been reported to foster the obesity-driven PCa progression through the secretion of different biofactors, including hormones, adipokines, and free fatty acids [29]. For instance, Laurent and colleagues have found that PCa cells can induce lipolysis in adipose tissue and that extracellular fatty acids are taken up by the tumor to support its own spread via activation of the NADP oxidase 5 (NOX5)/reactive oxygen species (ROS)/HIF1/MMP 14 pathway [30]. Furthermore, La Civita and coworkers have demonstrated that periprostatic adipocytes release TGF-β, which stimulates PCa cell motility through upregulation of the connective tissue growth factor (CTGF) [31]; the authors have also observed that the conditioned medium (CM) from adipose cells promotes disease resistance to chemotherapy through the release of IGF-1 and the subsequent overexpression of tubulin TUBB2B [32]. In our laboratory, we have highlighted that adipocyte CM endows castration-resistant PCa cells with stem-like properties, mesenchymal traits, and taxane insensitivity [33]. Despite these promising results, only a few studies have investigated the role of EVs in PCa-adipocyte interactions. In this regard, we have observed that adipose cell-derived sEVs can promote tumor aggressiveness by enabling multiple phenotypic and metabolic changes, including reduced docetaxel responsiveness and enhanced Akt/HIF-1α-dependent glycolysis [34]. These findings have later been confirmed by Mathiesen and colleagues, who have pointed out that the small vesicles from human adipose tissue explants increase the proliferation of metastatic PCa cells through TWIST1 modulation [35]. On the other hand, it has been recently evidenced that treatment of adipocytes with the antiandrogen receptor antagonist Casodex (i.e., Bicalutamide) induces their reprogramming toward a browning phenotype and that the EVs released from these brown-reprogrammed adipose cells significantly reduce PCa growth both *in vitro* and *in vivo*; this suggests that promoting the differentiation of white *vs*. brown adipocytes might represent a novel strategy to impair PCa development and progression [36]. Finally, Elmageed et al. have shown that PCa sEVs convert patient-obtained adipose stem cells toward a neoplastic state, by transferring their molecular cargo composed of pro-oncogenic mRNAs (H-Ras, N-Ras), a variety of oncomiRNAs (miR-125b, miR-155, miR-130b) as well as members of the Ras superfamily of GTPases (Rab1a, 1b and Rab11a) [37]. As previously mentioned, further investigations are needed to understand the molecular basis of the EV-mediated interaction network established between PCa and adipocytes within the TME.

### PCa-immune system interplay

In PCa microenvironment, a strict communication also exists between the tumor and infiltrating immune cells, such as T lymphocytes, macrophages, and natural killer (NK) cells [38]. This infiltrate collectively contributes to shaping the TME toward an immunosuppressive phenotype, now considered a common hallmark of cancers; it also participates in the secretion of proinflammatory/prometastatic cytokines as well as of enzymes involved in the remodeling of extracellular matrix (ECM) [38]. As illustrated in the following paragraphs of this review, accumulating evidence strongly supports the key role of EVs in the crosstalk between PCa and neighboring immune cells.

#### Interactions with T lymphocytes

T lymphocytes are the main mediators of the antitumor immune response and are the key target of checkpoint inhibitors, but they can also promote immune tolerance [39]. Intriguingly, it has been shown that sEVs from androgen-dependent PCa cells inhibit T lymphocyte proliferation, while triggering apoptosis; this immunosuppressive activity is mediated by the Fas ligand (FasL) expressed at their membrane level [40]. Similarly, sEVs derived from different cancer cell lines, including castration-resistant PCa cells, have been reported to impair T lymphocyte reactivity to interleukin-2, due to the presence of TGF-β1 on their surface [41]. These observations have later been corroborated by Sadovska and colleagues, who have observed that PCa small vesicles can directly interact with both B and T lymphocytes (CD3^+^ and CD8^+^) in a 3D heterotypic spheroid model of the tumor [42]. More importantly, numerous findings have revealed that programmed cell death ligand 1 (PD-L1), the main driver of cancer immune evasion, is located on the membrane of PCa sEVs [43, 44]. In this regard, Li and collaborators have highlighted that PD-L1-enriched small vesicles are secreted by hormone-unresponsive malignant cells and transferred to less aggressive PD-L1-negative tumor cells, thus protecting the latter from the cytotoxicity of T lymphocytes [45]. Likewise, Poggio et al. have found that castration-resistant cancer-secreted small vesicles carrying PD-L1 significantly impair T lymphocyte activation and favor tumor progression *in vivo*; notably, the injection of EV-deficient PCa cells, obtained by silencing two genes involved in vesicular biogenesis (*Rab27a* and *nSMase 2*), together with an anti-PD-L1 antibody has been associated with a remarkable decrease in cancer growth and a parallel increase in mouse survival [46].

#### Interactions with macrophages

Tumor-associated macrophages (TAMs) represent the main cellular components of the innate immune cell system in PCa microenvironment [38]. TAMs are commonly categorized in two functional subgroups: the M1 phenotype, endowed with proinflammatory and antitumoral activity, and the M2 phenotype, characterized by anti-inflammatory, protumoral, and immunosuppressive functions [47]. A polarization of TAMs from the M1 toward the M2 state is generally observed in cancers, and the key role of tumor-derived EVs in the modulation of the M1/M2 macrophage switch is now widely acknowledged [48, 49]. In the context of castration-resistant PCa, cancer-secreted sEVs have been shown to endow THP-1 monocytes with M2-like traits through the activation of the Akt and STAT3 pathways [50]. Likewise, Mezzasoma and coworkers have demonstrated that hormone-insensitive PCa EVs promote monocyte differentiation into active M2 macrophages that stimulate malignant migration [51]. Finally, Xu and colleagues have reported that androgen-independent PCa cells challenged with thapsigargin, a guaianolide able to deplete endoplasmic reticulum (ER) calcium stores and induce the ER stress-related unfolded protein response, can release small vesicles that induce macrophage M1-to-M2 transition, accompanied by the upregulation of PD-L1 and of multiple protumoral cytokines (IL-6, IL-10, and TGF-β) [52].

Based on the above observations, several investigations have been performed to address the role of PCa EV cargo in the communication with TAMs. Regarding the protein content of these vesicles, α_v_β_6_ integrin and chemokine (C-X-C motif) ligand 14 (CXCL14), highly expressed in metastatic PCa, have been shown to be transferred to monocytes and to confer them M2-like properties, thus contributing to tumor progression [53, 54]. Likewise, high mRNA levels of ring finger protein 157 (RNF157), a well-known E3 ubiquitin ligase which couples the PI3K and MAPK pathways with the cell cycle [55], have been detected in PCa sEVs, through which they are transmitted to macrophages; in these cells, RNF157 ubiquitinates the histone deacetylase-1 (HDAC1), initiating the M2 polarization process [56]. Similar results have been obtained in two recent studies exploring the role of miR-let-7 family, especially miR-let-7b and miR-let-7b-5p, in PCa immune evasion [57, 58]; on the other hand, Zhang and colleagues have recently reported that sEV-shuttled miR203 not only suppresses hormone-responsive PCa invasion *in vitro* and *in vivo* but it also triggers macrophage switch toward the M1 state, stimulating the accumulation of IL-1β, IL-6, IL-12, CXCL9, and CXCL10 in the TME and facilitating cancer eradication [59].

A new body of evidence has recently clarified the effects of TAM-shed EVs on tumor behavior [60]. In PCa, it has been found that sEV-packaged miR-95 from TAMs is taken up by malignant cells, where it interacts with the oncogenic transcription factor JunB and favors cancer proliferation, EMT, and invasion; in accordance with these data, increased miR-95 expression in PCa tissues correlates with worse clinicopathological features [61].

#### Interactions with other immune cells

PCa EVs are also known to affect the activity of natural killer (NK) cells, the main effectors in innate immunity; dendritic cells (DC), responsible for initiating antigen-specific immune responses; and myeloid-derived suppressor cells (MDSC), crucial mediators of immunosuppression. Specifically, Lu and coworkers have recently shown that circulating small vesicles in PCa patients can upregulate natural killer cell protein group 2A (NKG2A) in NK cells, thus blocking their cytotoxicity after prostatectomy [62]. It has also been observed that PCa sEVs inhibit the functions of DC cells through the transfer of prostaglandin E2 (PGE2) [63]. Finally, in an *in vivo* study, small vesicles from PCa have been demonstrated to promote the recruitment of MDSC cells into the TME, by increasing the expression of the chemokine receptor 4 (CXCR4) [64]; on the other hand, MDSC-derived sEV-carried S100A9, a calcium-binding protein with proinflammatory/protumoral properties, has been found to increase castration-resistant PCa aggressiveness via the circMID1/miR-506-3p/MID1 cascade [65].

### PCa-endothelium interplay

Angiogenesis, the formation of new blood vessels from preexisting ones, supplies cancer cells with oxygen, nutrients, and growth factors to support their proliferative, invasive, and metastatic behavior [66]. Of note, there is growing evidence that EVs regulate this process in different tumors, including PCa [67].

Integrins, the heterodimeric transmembrane receptors composing the cell-ECM adhesion structures, are known to play a pivotal role in endothelial cell proliferation and migration, thereby contributing to tumor angiogenesis [68]. Intriguingly, αvβ6 integrin has been shown to be upregulated in different malignancies, including primary and metastatic PCa [69]. Dr. Languino’s group has observed that elevated levels of this protein are expressed in PCa sEVs and are transmitted to αvβ6-negative tumor cells to boost their migratory potential [70]. More recently, the authors have also demonstrated that PCa vesicles that are uploaded with αvβ6 significantly increase the proliferation, junction formation, and tube forming capacity of human microvascular endothelial cells (HMEC1), supporting the key role of this integrin in tumor vascularization [71].

Leucine-rich α-2 glycoprotein 1 (LRG1) is a member of the leucine-rich repeat (LRR) protein family characterized by eight repeat sequences. It plays a crucial role in tumor evolution by favoring angiogenesis, based on its ability to enhance endothelial cell proliferation and invasion and to modulate the expression/activity of proangiogenic factors, such as TGF-β, angiopoietin-1, and VEGF-A [72]. In this regard, Liu and coworkers have demonstrated that LRG1 is overexpressed not only in castration-resistant PCa tissues but also in their small vesicles; remarkably, these particles are able to promote tube formation of human umbilical vein endothelial cells (HUVECs) *in vitro* [73].

Phosphoglycerate mutase 1 (PGAM1) is a key glycolytic enzyme that catalyzes the conversion of 3-phosphoglycerate into 2-phosphoglycerate. Of note, this protein has been reported to be involved in the mechanisms of cancer growth and metastasis [74]. Recently, it has been detected in metastatic PCa sEVs, by which it is carried to recipient HUVECs to promote their proliferative and angiogenic activity via γ-actin (ACTG1) remodeling [75].

Vesicular miRNAs have also been shown to be implicated in the angiogenic program occurring in PCa microenvironment. Specifically, Prigol and colleagues have highlighted that androgen-independent tumor-released small vesicles promote endothelial cell invasiveness and tube-forming efficiency via transfer of miR-27a-3p [76].

Although reports about the impact of EVs on PCa angiogenesis remain sparse, current data indicate that they might significantly influence the tumor-endothelium interactions.

## Biological roles of EVs in the horizontal communication between PCa cells

**Fig. 2:**
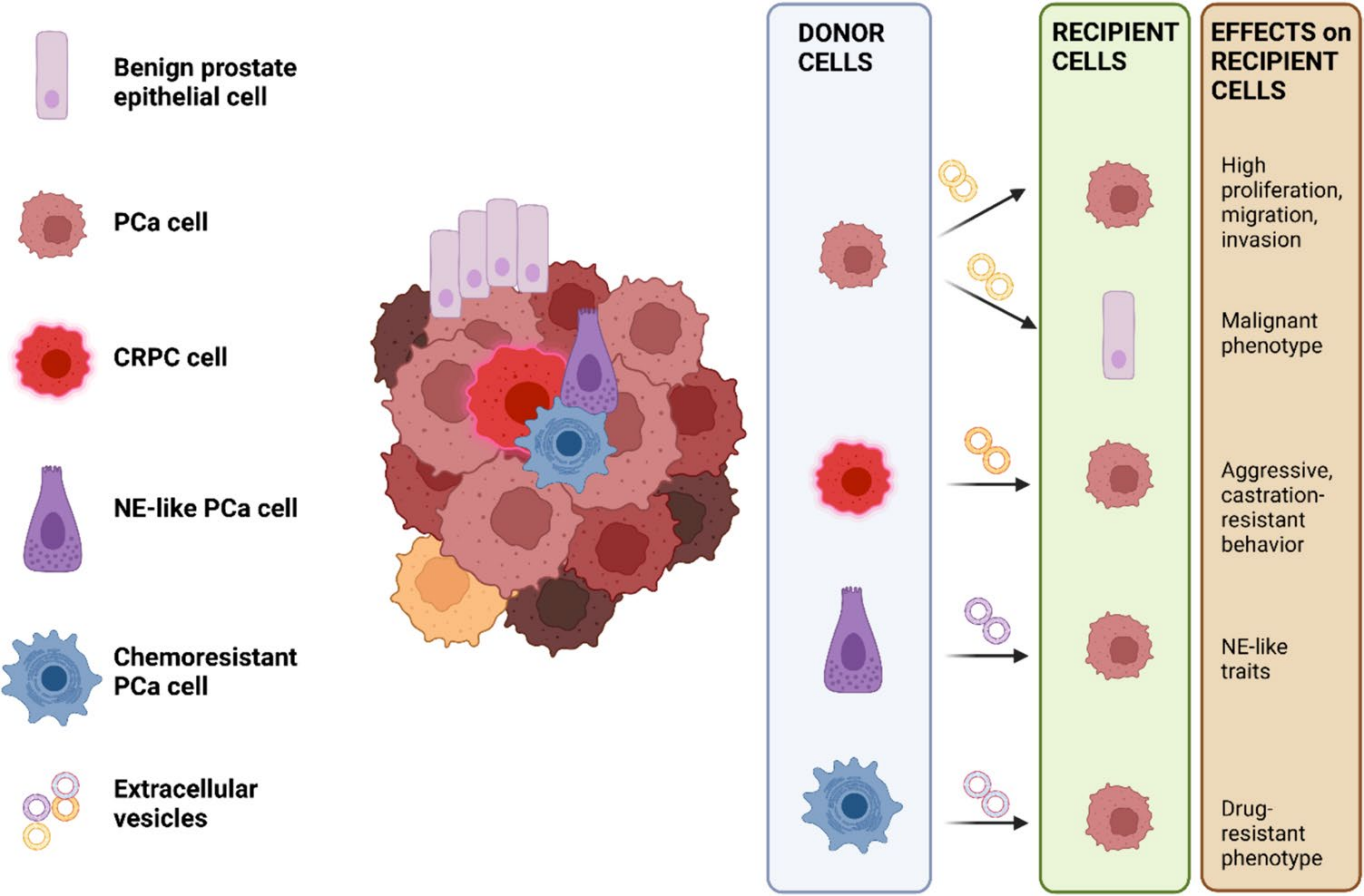
Schematic representation of the biological functions of EVs in the horizontal communication between PCa cells and surrounding non-cancerous and cancerous prostate cells. EVs released by PCa cells can induce tumorigenesis in surrounding benign prostate epithelial cells and promote the proliferation and invasion of other tumor cells. Moreover, PCa cells characterized by an aggressive (castration-resistant - CRPC, neuroendocrine-like - NE-like, or chemoresistant) phenotype release EVs able to transfer their peculiar malignant traits to neighboring cancer cells

PCa is characterized by a striking intratumor cellular heterogeneity and plasticity [77, 78]. In the last decade, numerous findings have highlighted the important role of PCa EVs in the remodeling of the characteristics of surrounding malignant and non-malignant prostate cells through the horizontal transfer of their molecular cargo (Fig. 2).

Hypoxia is commonly associated with poor prognosis in PCa [79]. Intriguingly, PCa cells grown in hypoxic conditions secrete increased numbers of sEVs that are taken up not only by surrounding stromal cells but also by adjacent epithelial cells in the tumor mass. In particular, it has been reported that vesicles from hypoxic tumor cells confer invasive and stem-like features to their normoxic counterpart; these effects are mediated by the activation of MMPs and enhanced synthesis of protumoral biofactors (TNFα, TGF-β, IL-6) as well as by a deep rearrangement in the epithelial adherens junctions [17].

EMT, the transition toward a mesenchymal phenotype, and NED, the transdifferentiation into a neuroendocrine-like state, represent crucial steps in PCa evolution [80–82]. Remarkably, EVs are profoundly implicated in the regulation of both these processes. For example, vesicles isolated from both solid and liquid biopsies of PCa patients have been found to educate normal prostate recipient cells toward a malignant invasive condition characterized by E/N-cadherin switch, vimentin overexpression, and IL-8 hyperproduction [83]. Moreover, sEVs from mesenchymal-like PCa cells have been shown to transmit plastic features, including increased migration and stemness, to their epithelial counterpart through the inhibition of AR, PSA, and ERG protein synthesis and the parallel activation of TGF-β pathway [84]. On the other hand, Lin et al. have demonstrated that sEVs secreted from enzalutamide-treated PCa cells are enriched with adipocyte differentiation-related protein (ADRP), which induces NED when horizontally transferred to neighboring tumor cells [85]. Parallelly, Patel and colleagues have found that suppression of miR-200c-3p correlates with upregulation of TBX2, a NED-related T-box transcription factor regulating SOX2 and N-MYC expression; of note, low levels of miR-200c-3p have been observed in small vesicles from TBX2-overexpressing PCa cells, allowing the propagation of neuroendocrine-like features across the entire tumor bulk [86].

EVs also play a key role in the lateral transmission of PCa drug resistance. Indeed, it has been reported that sEVs from castration-resistant cancer cells promote the conversion of androgen-sensitive cells into the aggressive hormone-independent phenotype through the activation of heme oxygenase-1 (HMOX1), a rate-limiting enzyme of heme degradation [87]. In addition, miR-222-3p has been observed to be exchanged between castration-unresponsive and responsive PCa cells via small vesicles, favoring the dissemination of drug tolerance by activating mTOR signaling through targeting of midnolin [88]. In line with these observations, Corcoran and coworkers have highlighted that sEVs from docetaxel-resistant PCa cells are able to confer chemotherapy insensitivity to parental tumor cells, partly due to their MDR-1-enriched molecular cargo [89]. Conversely, it has been pointed out that EVs produced by non-cancerous prostate epithelial cells can revert drug resistance in camptothecin- and paclitaxel-unresponsive malignant cells, suggesting that they may serve as useful tools for the management of the most aggressive forms of PCa [90].

Long non-coding RNAs (lnc-RNAs) are ncRNAs defined by their length (more than 200 nucleotides) and grouped in different subclasses according to their biogenesis, function, and structure. They lack protein-coding activity and act as regulators of gene expression, thereby participating to several physiological and pathological processes, including cancer [91]. Remarkably, different lncRNAs have been shown to be dysregulated in PCa [92, 93]. In particular, Wang and coworkers have reported that the lncRNA MYU is overexpressed in PCa tissues, where it serves as a tumor-promoting factor. Castration-resistant PCa cells overexpressing MYU release sEVs loaded with high levels of this lncRNA, which is transferred to naїve tumor cells to boost their proliferative and migratory ability; mechanistically, MYU binds to miR-184 and upregulates c-Myc expression [94]. Similarly, it has been demonstrated that the lncRNA PCSEAT (PCa-specific expression and EZH2-associated transcript) is upregulated in hormone-independent malignant cells and is endowed with oncogenic properties. Specifically, PCSEAT promotes PCa cell growth by sponging miR-143 and miR-24–2-5p, thus regulating the expression of the histone methyltransferase EZH2 (polycomb group protein enhancer of zeste homolog 2). Intriguingly, PCSEAT-negative cancer cells treated with small vesicles from their PCSEAT-overexpressing counterpart exhibit a highly aggressive phenotype [95]. Finally, it should be emphasized that sEV-carried lncRNAs also participate to the horizontal communication between androgen-independent and androgen-dependent PCa cells. Indeed, Jiang and coworkers have reported that the lncRNA HOXD-AS1 is highly present in hormone-insensitive cells as well as in their vesicles; when taken up by hormone-responsive cells, vesicular HOXD-AS1 significantly increases tumor migration, by inducing E-cadherin downregulation and vimentin upregulation [96].

Circular RNAs (circRNAs) are a novel class of single-stranded ncRNAs that lack the canonical 5′ end cap and 3′ end poly (A) tail. Unlike linear RNAs, in circRNAs the 5′ and 3′ ends are joined together to form a covalently closed continuous loop. These features confer them specific biological properties and functions. For instance, circRNAs have been observed to act as miRNA sponges and key regulators of gene transcription or splicing; moreover, some of them are endowed with protein-coding functions. Recent research has revealed a dysregulation of circRNAs in different pathologies, including cancer [97, 98]. In PCa, different circRNAs have been found to be either upregulated or downregulated in tumor tissues, where they promote cancer growth, metastasis, and drug resistance [99]. In this setting, it has been evidenced that sEVs from PCa patients are enriched with circKDM4A; treatment of PCa cells with these particles increases their proliferation and migration, while inhibiting apoptosis. Mechanistically, circKDM4A exerts its protumoral effects by sponging miR-338-3p, thereby leading to the overexpression of cullin 4B (CUL4B), an ubiquitin ligase known to be endowed with oncogenic properties [100]. Similar observations have been reported for circHIPK3 and circ-0081234, found to be overexpressed in small vesicles from both the serum of PCa patients and supernatant of PCa cells and to stimulate the growth and invasion of recipient tumor cells via miR-212/B-cell-specific MMLV insertion site-1 (BMI-1) and miR-1/MAP3K1 axis, respectively [101, 102].

Even though the results reviewed in this chapter are intriguing, additional studies are required to fully clarify the biological involvement of EVs in PCa drug resistance.

## Clinical application of EVs as diagnostic and prognostic biomarkers for PCa

Together with digital rectal examination and transrectal ultrasound scan, screening of serum prostate-specific antigen (PSA) is commonly used for the detection of PCa [103, 104]. However, as this glycoprotein is expressed in both tumor and non-malignant cells, it remains tissue-specific and is not pathology-sensitive. In particular, PSA levels can be altered in various non-cancerous conditions, including prostatitis, urinary tract infection and benign prostate hyperplasia (BPH), often leading to overdiagnosis and overtreatment. Indeed, out of the men displaying elevated PSA concentrations in the blood, only 25% are diagnosed with PCa. To precisely correlate an increase in PSA levels with malignant diseases, other aspects are generally taken into consideration, such as the ratio between total PSA, free PSA, and pro-PSA (prostate health index test) or the combination of kallikrein-related peptidase 2 (hK2), intact PSA, free PSA, and total PSA (4-kallikrein test). Nonetheless, although these assays allow a better distinction between benign and malignant prostate gland hypertrophy and have significantly improved the prediction of high-grade and clinically aggressive tumors, their accuracy is still suboptimal [103, 104]. In this context, EV molecular cargo has been widely analyzed to find novel and more specific PCa biomarkers, with many studies demonstrating that EV-derived DNAs, RNAs, proteins, and metabolites are able to discriminate between healthy and pathological conditions, distinguish different disease stages, and predict tumor prognosis or treatment efficacy (Table 1).

**Table 1:** Table 1

Marker type	Marker name	Sample	Role	Ref
DNA	Genomic DNA	Serum	Diagnostic/prognostic	[105–107]
mRNA	TMPRSS2:ERG	Urine	Diagnostic	[108–110]
	AGR2-SV-G and SV-H	Urine	Diagnostic	[111]
	CDH3	Urine	Diagnostic	[112]
	AR and AR-V7	Urine and serum	Prognostic	[113–115]
	BRN2 and BRN4	Serum	Prognostic	[116]
miRNA	miR-141, miR-375 and miR-21	Urine and serum	Diagnostic/prognostic	[117–122]
	miR-1290	Serum	Diagnostic/prognostic	[118, 123]
	let-7a	Urine and serum	Diagnostic/prognostic	[121, 122]
	miR-34a	Serum	Prognostic	[124]
	miR-654 and miR-379	Serum	Prognostic	[125]
	miR-19b, miR-130b, miR-145,	Urine and serum	Diagnostic/prognostic	[126]
	miR-196a, miR-200c, miR-501,			
	miR-521, miR-572, miR-574,			
	miR-885, miR-1246 and miR-2909			
lncRNA	lincRNA-p21	Urine	Diagnostic	[127]
	linc00662, CHASERR and lnc-LTBP3-11	Urine	Diagnostic	[128]
	SAP30L-AS1 and SChLAP1	Serum	Diagnostic	[129]
Protein	Filamin A	Serum	Diagnostic/prognostic	[130]
	PSA and PSMA	Urine and serum	Diagnostic/prognostic	[131–134]
	PD-L1, ERG, Integrin-β5, Survivin, TGF-β, p-TSC2, MAPK and mTOR	Serm	Diagnostic	[135]
	ADSV-TGM4 and CD63-GLPK5-SPHM-PSA-PAPP	Urine	Diagnostic/prognostic	[136]
	SERPINA3, LRG1 and SCGB3A1	Urine	Diagnostic	[137]
	TM256 and LAMTOR1, VATL, ADIRF	Urine	Diagnostic	[138]
	CD5 antigen-like protein, complement C1q C Chain, leucine-rich alpha-2-glycoprotein, pregnancy zone protein, haptoglobin and inter-alpha-trypsin inhibitor heavy chain H2	Urine and serum	Prognostic	[139]
	LRG1	Serum	Prognostic	[140]
	P-gp and ABCB4	Serum	Prognostic	[141, 142]
	Fructose-bisphosphate aldolase, cytosolic nonspecific dipeptidase, CD63, CD151, myosin light chain 9 and peroxiredoxin-6	Serum	Prognostic	[143]
Lipid	Phosphatidylserine and lactosylceramide	Urine	Diagnostic	[144]
	Phosphatidylglycerol and diacylglycerol	Urine	Diagnostic	[145]
	Arachidonic acid, ceramides, phosphathidylcholines and acyl carnitines	Urine	Diagnostic	[146]

### DNA-based biomarkers

Cancer cell-free DNA (cfDNA) represents a fraction of circulating DNA that is released into the bloodstream from dying tumor cells. It reflects the genetic and epigenetic status of the tumor of origin, displaying the same mutations, copy number alterations, chromosomal rearrangements, and methylations. Thus, cfDNA analyses can be employed to track intratumoral heterogeneity, disease progression, and therapy response [147]. However, the amount of cancer cfDNA within the entire circulating DNA may be very low, and ultrasensitive technologies, including digital-droplet PCR, BEAMing, and tagged amplicon sequencing, are required to detect rare tumor variants. In addition, some of the genetic alterations in the cfDNA may be due to the age-related clonal expansion of mutated hematopoietic cells. Remarkably, emerging evidence indicates that the DNA molecules present in plasma EVs show superiority over cfDNA as cancer biomarkers [148]. Indeed, Balaj and coworkers have recently examined the content of the lEVs derived from PCa patients, showing that they contain both genomic and complementary DNA similar to the parental tumor [149]. Likewise, Casanova-Salas and colleagues have found that sEV DNA features recapitulate matched-patient biopsies, also associating with clinical progression [150]. Finally, Dr. Lazaro-Ibanez’s group has demonstrated that the genomic DNA content differs across PCa EV subtypes, suggesting a possible selective packaging of DNA into distinct vesicular compartments [105]; this notion has been later confirmed by Vagner et al., who have shown an enrichment of genomic DNA in plasma lEVs compared to smaller vesicles [106]. Collectively, these studies warrant further research for the use of vesicular DNA as an additional biomarker for diagnosis, prognosis, and management of PCa.

### RNA-based biomarkers

Full-length or fragmented mRNAs have been reported to be carried by cancer-released EVs, including those from PCa [141]. The molecular content of the vesicles isolated from the urine or the blood has already been validated as a source of prostate-specific mRNAs, such as the oncogenic fusion gene TMPRSS2:ERG [107, 151]. In particular, the TMPRSS2:ERG transcript found in urinary EVs has been shown to display a significant predictive value when combined with ERSPC (European Randomised Study of Screening for Prostate Cancer) risk calculator, thus exhibiting great potential for limiting the number of unneeded prostate biopsies [108]. Additional mRNAs, such as CDH3, are downregulated in urine-derived particles of PCa patients with respect to men with BPH, while others, including urinary EV AGR2-SV-G and SV-H, display even a better accuracy than PSA in PCa diagnosis [109, 110]. Importantly, AR full-length and AR-V7 transcripts have been detected in both plasma and urine small vesicles from metastatic patients, predicting the emergence of a condition of castration resistance [111–113]. Likewise, the mRNAs of BRN2 and BRN4, which are transcription factors commonly associated with neuroendocrine features of PCa, have been identified in circulating sEVs [114]. Despite the function that vesicular mRNAs might play within biofluids or recipient tissues, these numerous findings designate the transcript cargo of EVs as an intriguing source of PCa biomarkers.

miRNAs are the largest RNA population in most of the EVs, thus representing ideal diagnostic and prognostic candidates [142]. Among all the PCa EV miRNA markers, miR-141, miR-375, and miR-21 are the most investigated and demonstrate consistent trends across various studies. Indeed, they are upregulated in tumor small vesicles from both serum and urine and are crucially implicated in fundamental events of cancer progression, such as metastasis and castration resistance [115–119, 152]. In addition to these molecules, miR-1290 has been reported to be overexpressed in plasma sEVs of patients with PCa compared to those with BPH and to strictly correlate with poor prognosis [116, 120]. Similarly, let-7a shuttled by plasma small vesicles could be used to distinguish PCa patients with GS ≥ 8 from those with GS ≤ 6, while its presence in urine significantly differs in PCa patients and healthy controls [118, 119]. Finally, circulating sEV-carried miR-654 and miR-379 have been demonstrated to predict radioresistance, and decreased miR-34a levels have been associated with docetaxel insensitivity [121, 122]. Other EV miRNAs endowed with diagnostic and prognostic potential are miR-19b, miR-130b, miR-145, miR-196a, miR-200c, miR-501, miR-521, miR-572, miR-574, miR-885, miR-1246, and miR-2909 [123].

Recently, a prostate-specific lncRNA, the PCa gene 3 (PCA3), has been approved as an additional test to determine the need for biopsies in PCa. Based on these premises, many studies have been conducted to characterize the lncRNA content of tumor-secreted EVs. In this regard, Işin et al. have highlighted the diagnostic utility of urine-derived sEV-packaged lincRNA-p21 in men with PCa [124]. Likewise, linc00662, CHASERR, and lnc-LTBP3-11 contained in urinary small vesicles have been identified by Bajo-Santos and coworkers as PCa biomarkers [125]. Finally, Wang and colleagues have shown that plasma levels of sEV SAP30L-AS1 are upregulated in BPH, whereas sEV SChLAP1 is significantly overexpressed in PCa individuals; in particular, SAP30L-AS1 expression correlates with PSA values and tumor invasion, while SChLAP1 levels are associated with Gleason score, also helping to differentiate between BPH and PCa when the concentration of PSA is in the gray zone [126]. Altogether, these data suggest that the discriminative potential of EV lncRNAs may improve the diagnostic prediction of the malignant state for patients affected by PCa.

### Protein-based biomarkers

African American men exhibit the highest PCa incidence and mortality rates compared to other races, making this tumor a challenging racial disparity disease. In this setting, serum EVs have been recently demonstrated to contain ethnically specific biomarkers that could be exploited for their diagnostic and therapeutic potential. Specifically, 22 sEV-carried proteins have been found to be unique to the African American ethnicity; among them, filamin A has been proposed as a novel serum small vesicle-based “protein signature” for potential use in the early detection and better prognosis of PCa in African American patients [127, 143].

As reported above, both PSA and PSMA plasma levels have important diagnostic and prognostic value. Remarkably, these proteins have been found to be present in plasma EV samples from PCa patients, while not being detected in healthy men [128, 129, 153]. In particular, serum PSMA-positive EV concentration has been successfully used to differentiate BPH from PCa and to distinguish tumors with GS ≤ 7 from those with GS ≥ 8 [128, 129]. Of note, Mitchell et al. have demonstrated that PSA and PSMA are also expressed in the urinary small vesicles of a cohort of 24 PCa patients [130]. Taken together, these data clearly indicate that both plasma and urinary vesicular compartments hold promise as non-invasive sources of cancer-associated antigens.

In addition to well-known PCa markers, recent studies have been focused on the identification of new molecular candidates for tumor diagnosis and prognosis. Among others, PD-L1, ERG, integrin-β5, survivin, TGF-β, and phosphorylated-TSC2 as well as partners of the MAP-kinase and mTOR pathways have emerged as differentially expressed endpoints in tumor-derived EVs [131]. Similarly, Sequeiros and collaborators have identified a two-protein combination in urinary small vesicles that classifies benign and PCa patients (ADSV-TGM4) and a combination of five proteins able to significantly distinguish between high- and low-grade PCa individuals (CD63-GLPK5-SPHM-PSA-PAPP) [132]. Likewise, 18 sEV proteins have been reported to be differentially expressed in PCa, three of which (SERPINA3, LRG1, and SCGB3A1) have been shown to be consistently upregulated and to add predictable value in addition to age, prostate size, body mass index (BMI), and PSA [133]. Moreover, TM256 and LAMTOR1 have been observed to be overexpressed in men affected by PCa compared to healthy male controls, displaying high sensitivity and specificity for the tumor both as individual biomarkers and in combination with each other [134]. Other prominent proteins are V-type proton ATPase 16-kDa proteolipid subunit (VATL), adipogenesis regulatory factor (ADIRF), and several Rab-class members and proteasomal molecules [134]. Finally, Dhondt and colleagues have found hundreds of previously undetected proteins in the urinary small vesicles of PCa subjects, providing a powerful toolbox to map uEV content and contaminants [154].

As previously illustrated, castration resistance represents a huge problem in PCa evolution. Integration of proteomics and metabolomics data has generated molecular signatures of plasma sEVs that may facilitate discrimination of CRPC from PCa and tumor-free men. In particular, six vesicular proteins, namely, CD5 antigen-like protein, complement C1q C Chain, leucine-rich alpha-2-glycoprotein, pregnancy zone protein, haptoglobin, and inter-alpha-trypsin inhibitor heavy chain H2, have been exclusively found in both serum and urine of CRPC patients, while not being present in control samples [135]. More importantly, it has been recently highlighted that the sEV protein LRG1 plays a crucial role in PCa malignant progression, suggesting that it could serve as a predictive indicator of castration resistance [73].

Taxanes, a class of microtubule-targeting anticancer agents, are currently used as the first-line chemotherapy for PCa. However, drug resistance develops in 40% of cases [136, 144]. In order to predict or monitor the emergence of chemoresistance, several studies have been investigating EV protein cargo. In this context, P-glycoprotein (P-gp) and ABCB4, which act as drug efflux pumps and contribute to reduced chemotherapy response [145, 146], have been found to be overexpressed in clinically docetaxel-resistant patients compared to treatment naïve individuals [137, 138]. On the other hand, fructose-bisphosphate aldolase, cytosolic nonspecific dipeptidase, CD63, CD151, myosin light chain 9, and peroxiredoxin-6 were elevated in EVs from both cabazitaxel-resistant cell lines and patients, with PI3K signaling activation being responsible for the development of chemoresistance [155].

### Lipid-based biomarkers

Lipids play pivotal functions in cells, including energy storage, provision of structural support of biological membranes, and signaling. All these cellular processes are critically relevant for cancer development and progression, with specific classes of lipids being reflective of tumor stage [156]. Interestingly, Skotland and colleagues have analyzed the lipidome of urinary sEVs in 15 PCa patients and 13 healthy controls and have found that the levels of nine lipid species significantly differ between the two groups [139]. The highest significance is shown for phosphatidylserine (PS) 18:1/18:1 and lactosylceramide (d18:1/16:0), and the combinations of these lipids and PS 18:0–18:2 distinguish the two groups with 93% sensitivity and 100% specificity. A similar analysis has also been conducted by Yang and collaborators, who have found an increase in phosphatidylglycerol (PG) 22:6/22:6 and a significant decrease in (16:0,16:0)- and (16:1, 18:1)-diacylglycerol (DAG) species in men with PCa compared to healthy controls [140]. Finally, Clos-Garcia et al. have identified 76 metabolites from urinary EVs that exhibit significant differential abundance between PCa and BPH, such as arachidonic acid, ceramides, phosphatidylcholines, and acyl carnitines [157]. Overall, these studies highlight that lipidomic analysis of urinary EVs has great potential for the discovery of new biomarkers of PCa.

## Conclusion and future directions

PCa is a major health problem with an urgent need for the identification of biomarkers for the early diagnosis of the disease, the screening of populations at higher risk, and the prognostic evaluation of patients’ response to treatments. To this purpose, the mechanisms of PCa onset and evolution have been largely investigated. In particular, a growing body of evidence has raised the attention on the role of EVs in PCa development and progression. Of note, the use of new technologies has significantly contributed to the characterization of the transcriptomic, proteomic, and metabolomic cargo of PCa vesicles and has deepened our understanding of the TME and of the molecular cascades implicated in disease pathogenesis. More importantly, despite the challenges related to the identification of clinically valuable biomarkers for tumors, the numerous clinical trials conducted so far on PCa EVs have revealed their great potential for improving diagnostic accuracy, prognostic outcome, and treatment response. Nonetheless, EV heterogeneity still represents one of the major challenges for biomarker discovery, with current bulk population-based methods for vesicle isolation and classification often failing to provide clinically relevant information; analysis of EVs at the single particle level through digital assays, flow cytometry, nanoparticle tracking analysis, Raman spectroscopy, or microscopy techniques may hold the key to the development of personalized medicine. Hopefully, the newly identified biomarkers will apply to larger subgroups of patients, thus substantially promoting a reduction in the incidence of the malignancy. This could also result in enormous advances in the management of metastatic or resistant tumors, by preventing disease relapse and facilitating the selection of more specific and effective therapies. Last but not least, it will be interesting to see if liquid biopsies could gradually replace more invasive procedures.

## Data Availability

No datasets were generated or analysed during the current study.
